# Neuropilin 1 Regulation of Vascular Permeability Signaling

**DOI:** 10.3390/biom11050666

**Published:** 2021-04-29

**Authors:** Alison Domingues, Alessandro Fantin

**Affiliations:** Department of Biosciences, University of Milan, Via G. Celoria 26, 20133 Milan, Italy

**Keywords:** neuropilin 1, permeability, endothelium, VEGFA, semaphorin

## Abstract

The vascular endothelium acts as a selective barrier to regulate macromolecule exchange between the blood and tissues. However, the integrity of the endothelium barrier is compromised in an array of pathological settings, including ischemic disease and cancer, which are the leading causes of death worldwide. The resulting vascular hyperpermeability to plasma molecules as well as leukocytes then leads to tissue damaging edema formation and inflammation. The vascular endothelial growth factor A (VEGFA) is a potent permeability factor, and therefore a desirable target for impeding vascular hyperpermeability. However, VEGFA also promotes angiogenesis, the growth of new blood vessels, which is required for reperfusion of ischemic tissues. Moreover, edema increases interstitial pressure in poorly perfused tumors, thereby affecting the delivery of therapeutics, which could be counteracted by stimulating the growth of new functional blood vessels. Thus, targets must be identified to accurately modulate the barrier function of blood vessels without affecting angiogenesis, as well as to develop more effective pro- or anti-angiogenic therapies. Recent studies have shown that the VEGFA co-receptor neuropilin 1 (NRP1) could be playing a fundamental role in steering VEGFA-induced responses of vascular endothelial cells towards angiogenesis or vascular permeability. Moreover, NRP1 is involved in mediating permeability signals induced by ligands other than VEGFA. This review therefore focuses on current knowledge on the role of NRP1 in the regulation of vascular permeability signaling in the endothelium to provide an up-to-date landscape of the current knowledge in this field.

## 1. Introduction

The vascular system consists of a complex network of blood vessels organized as a closed circulatory system in all vertebrates as well as some invertebrates [1,2]. The vascular system carries blood through all the districts of the organism to deliver oxygen and nutrients, which are necessary for organ and tissue homeostasis, and to remove waste and catabolites. Therefore, it does not surprise that the vascular system is the first organ system to form in the developing vertebrate embryos [3,4], at a time when blood vessels also contribute to primitive hematopoietic development [5,6]. Moreover, circulating immune cells interact with blood vessels to extravasate and provide immunosurveillance and establish innate or adaptive immunity in pathological conditions [7].

## 2. Vascular Permeability

The inner lining of all blood vessels is formed by a monolayer of endothelial cells (ECs) that are anchored to a basement membrane on the abluminal side and joined together by intercellular junctional complexes. The primary function of the vascular endothelium is to serve as a selective barrier between the blood and each tissue in the body, whereby the permeability of the endothelium to blood cells, plasma macromolecules and water can be adapted according to the physiological need and localization. For instance, blood vessels in the kidney and in endocrine organs show a high basal permeability to enable plasma filtration and hormone release into the bloodstream, respectively. In contrast, the blood-brain barrier forms a tight, highly impenetrable interface to maintain the central nervous system in a more protected environment [8].

Vascular permeability, i.e., the movement of solutes and molecules from the luminal to the abluminal side of the endothelial barrier, can be modulated by the exposure to permeabilizing agents. However, certain molecules can cause the permeability of the vascular endothelium to become excessive, resulting in acute or chronic vascular leakage. Such vascular hyperpermeability contributes to the pathophysiology of several human disorders, including cancer, heart, brain and limb ischemia, neovascular eye diseases and chronic inflammatory conditions [9,10,11,12].

## 3. Vascular Permeability in Pathology

Vascular permeability can be beneficial after acute tissue injury through the delivery of coagulation factors, antibodies and cytokines. However, the leakage of plasma molecules during chronic hyperpermeability can cause pathological tissue edema, which is the accumulation of fluids in the extracellular space that induces deleterious swelling and increases interstitial pressure. Moreover, vascular hyperpermeability can foster leukocyte recruitment, which favors inflammation, often promoting disease progression (reviewed by [12]). In addition, vascular hyperpermeability is recognized as a cardinal feature of newly formed blood vessels in those diseases characterized by an expansion of the vasculature, usually abnormal or disorganized, by a process called pathological angiogenesis [8].

In cancer, disruption of the vascular barrier may potentiate tumor cell intravasation and/or extravasation, leading to widespread metastatic disease, while increased interstitial pressure often prevents efficient drug delivery to cancer sites (reviewed by [12]). Moreover, tumor angiogenesis results in cerebral edema in glioblastoma multiforme, and in ascites and pleural effusions in liver metastasis and lung cancer, respectively [13,14,15]. In ophthalmic diseases, such as the proliferative form of diabetic retinopathy that leads to diabetic macular edema (DME) and the wet form of age-related macular degeneration (AMD), abnormal vessel growth and increased vascular permeability promote retinal edema, which disrupts neural function and subsequently results in visual loss (reviewed by [16]). Vascular hyperpermeability may also contribute to increased lipid deposition in atherosclerosis, resulting in neointimal hyperplasia [17]. Furthermore, the acute phase of ischemic events, such as myocardial infarction, is accompanied by edema contributing to tissue damage and disease outcome [18].

Stimulating blood vessel growth through angiogenesis is considered a promising treatment for organ ischemia and may provide a useful method to increase delivery of therapeutics to poorly perfused tumors. However, any beneficial effect of supportive angiogenesis will be hampered if accompanied by edema generation. This is unfortunately the case for the most potent angiogenic factor described to date, the vascular endothelial growth factor A (VEGFA), whose expression is associated with re-vascularization of damaged tissues but also increases vascular permeability. In fact, VEGFA was originally identified because of its potent permeability-inducing properties and accordingly first named vascular permeability factor (VPF). After two decades of VEGFA research, it is still not clear how VEGFA and its receptors selectively induce vessel growth versus vascular permeability to meet specific physiological needs, and how excessive vascular permeability may be controlled to limit tissue damage caused by edema. For these reasons, any novel insight on the signaling pathways that modulate different vascular responses, for example to attenuate vascular leakage in ischemic diseases without preventing new vessel growth, will be of fundamental value for devising more efficient therapeutic interventions. This review will therefore focus on the cellular mechanisms mediating vascular permeability and, in particular, the latest updates on the molecular mechanisms by which the VEGFA co-receptor neuropilin 1 (NRP1) modulates vascular responses to regulate permeability in both physiological and pathological settings.

## 4. Cellular Mechanisms of Vascular Permeability

Vascular permeability occurs via paracellular or transcellular routes [8,19,20]. Paracellular permeability describes the flow of fluid and solutes through the space between endothelial cells, a process that is regulated by cell-cell junctional complexes. Endothelial cell-cell junctions are assembled by a series of adhesion molecules that make up tight and adherens junctions [21]. Both tight and adherens junctions are formed by transmembrane proteins that generate a zipper-like structure along the cell border and mediate adhesion to the adjacent cell. As the name suggests, tight junctions are the tightest and their major transmembrane constituents that mediate intercellular interactions include claudins, the junction-associated molecule (JAM) family and occludin, which exist in complex with intracellular scaffold proteins such as cingulin, paracingulin and zona occludens (ZO) family members. Adherens junctions, instead, are mainly composed by vascular endothelial (VE)-cadherin (CDH5), which is a single-span transmembrane protein exclusively expressed by endothelial cells and its extracellular domain forms homomeric dimers with VE-cadherin molecules of adjacent cells. Weakening of VE-cadherin-mediated cell-cell junction is triggered by tyrosine and serine phosphorylation of both cadherins and their intracellular interactors, such as β-catenin, which results in internalization of the complex. Ultimately, barrier function is affected by the disruption of the protein bridge linking adherens junctions to the actin cytoskeleton [22,23]. Junctions are dynamically remodeled to control vascular permeability and loss of junctional integrity increases both the amount of paracellular leakage as well as the size of the macromolecules that are allowed to cross the barrier. Indeed, vascular hyperpermeability in response to permeability-inducing agents, such as VEGFA, have long being associated to a reduced expression shown by staining for junctional proteins such as ZO1 [24], occludin [25] or VE-cadherin [25,26,27,28] at the endothelial junctions.

Transcellular permeability describes the transfer of fluid and solutes through the cell from the apical to basal side of the endothelium (or viceversa) via vesicles, e.g., caveolae or vesicles complexes fusing into transendothelial channels such as the vesiculo-vacuolar organelle (VVO) [29]. However, due to the requirement of electron microscopy and the lack of definitive molecular markers or loss-of-function models, the study of this permeability pathway has proven particularly challenging. So far, only caveolin 1, the signature protein of endothelial cell caveolae, has been proven necessary for the regulation of VVO function, but not VVO structure, in acute vascular hyperpermeability [30]. Finally, another ultrastructural feature regulating the passage of macromolecules is represented by fenestrae, also called fenestrations, within ECs that facilitate rapid transport across the endothelium in endocrine tissues or organs specialized in blood filtration. Fenestrae are small pores that, depending on the tissue, can be covered by a diaphragm composed by plasmalemma vesicle associated protein (PLVAP) [31].

## 5. NRP1: Structure

The NRPs are a family of single pass transmembrane proteins of about 130 kDa. In mammals and most vertebrates, two NRP family members exist, NRP1 and NRP2, which share the same overall domain structure and are, on average, 44% identical at the amino acid level [32]. In zebrafish, instead, genome duplication in a teleost ancestor resulted in the presence of 4 members, nrp1a, nrp1b, nrp2a and nrp2b [33].

All NRPs are composed of a relatively large extracellular portion, a short transmembrane domain and a cytoplasmic domain of 43-44 amino acids [34,35,36,37]. NRP1 extracellular region includes five domains: a1, a2, b1, b2 and c. The a1 and a2 domains bind the core seven-bladed Sema domain of class 3 semaphorins, [38,39], while the b1 and b2 mediate binding to VEGFA, the basic tail of semaphorins and heparin and they additionally promote cell adhesion [40,41] (we refer to the next chapter for a more detailed description of NRP1 ligands). The c and the transmembrane domains are involved in receptor dimerization, whereas the cytoplasmic tail does not contain a signaling domain but a PDZ (PSD-95/Dlg/ZO-1) binding-motif with a SEA amino acid triplet at the carboxy terminus that allows the formation and stimulation of signaling complexes. Alternative splicing events can also produce soluble forms of both NRP1 and NRP2 (sNRP1, sNRP2) or an isoform of NRP2 without a SEA motif [42].

## 6. NRP1: Molecular Function and Ligands

**Figure 1:**
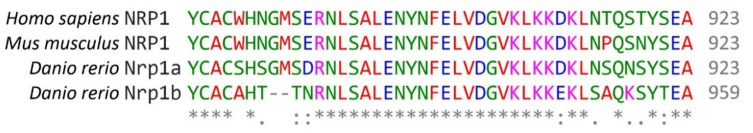
Alignment of the C-terminal amino acid sequence of human NRP1, mouse NRP1 and zebrafish Nrp1a and Nrp1b, including the complete cytoplasmic domain. Alignment performed with Clustal Omega (European Bioinformatics Institute; EBI). Asterisks indicate positions at which residues are conserved in all three species; colons and period indicate residues that are semi-conserved, i.e., have strongly or weakly similar properties, respectively. Color coding: red, small and hydrophobic residues; blue, acidic residues; magenta, basic residues; green, hydroxyl, sulfhydryl and amine residues and glycine.

NRP1 is able to form homodimers or heterodimeric complexes with NRP2 [43], even though genetic studies showing the requirement of NRP1 but not NRP2 in angiogenesis [38,44] and vascular permeability [45] suggest that it does not mainly function as a heterodimer in endothelial cells. NRP1 acts primarily as a co-receptor, binding secreted ligands and forming complexes with the ligand-specific receptors that promote downstream signaling, e.g., vascular endothelial growth factor receptors (VEGFRs) for VEGFA and plexins for class 3 semaphorins. Despite the highly conserved amino acid sequence of the NRP1 cytoplasmic tail across species, which suggests an essential role for this domain (Figure 1), NRP1 lacks an intracellular catalytic activity and is generally considered not to possess intrinsic signaling capabilities [46], although a few reports seem to indicate that its cytoplasmic tail can signal independently of other receptors [47,48]. Instead, the short intracellular domain of NRP1 acts by recruiting proteins to the cytoplasmic side of NRP1-containing receptor complexes. For example, it binds synectin, a PDZ-domain protein, also called GIPC1 (GAIP-interacting protein C terminus, member 1), to enhance VEGFA signaling in ECs and promote VEGFA-induced arteriogenesis [49,50,51,52,53].

NRP1 has been widely studied as a receptor for the secreted glycoprotein VEGFA. VEGFA gene contains 8 exons and, judging from transcript levels, is expressed as three main isoforms that differentially include exons 6 and 7 [54,55]. In humans, these isoforms are termed VEGFA121, VEGFA165 and VEGFA189 to reflect the number of amino acids in each isoform after subtraction of the 26 amino acid long signal peptide (total 147, 191 and 215 amino acids, respectively). Each isoform in mice is one amino acid shorter. The protein domains encoded by exons 6 and 7 provide VEGFA with affinity for the extracellular matrix, which in turn affects the diffusibility of each isoform. Thus, VEGFA121 and VEGFA189 are the most and the least diffusible among the major isoforms, respectively, whilst VEGFA165, whose mRNA is the most abundant in most organs [54,55], shows intermediate properties [56]. The differential distribution of each isoform in the extracellular space and the formation of chemotactic gradients is critical for normal vascular morphogenesis [57]. Moreover, VEGFA isoforms show distinct receptor binding properties. Thus, all the isoforms can bind the two main VEGFA tyrosine kinase receptors, VEGFR1 (FLT1) and VEGFR2 (KDR, previously also known as FLK1), while NRP1 binds with higher affinity the larger VEGFA isoforms, such as VEGFA165 and VEGFA189, compared to VEGFA121 by interacting with the heparin binding domain encoded by exon 7, even though the interaction between VEGFA and NRP1 also involves the exon 8 encoded epitope, which is common to all the isoforms [58,59,60,61]. NRP1 has also been reported to interact with other ligands that share homology with VEGFA, such as VEGFB, VEGFC, VEGFD and the placental growth factor 2 (PLGF2, also known as PGF), as well as other heparin-binding growth factors, such as hepatocyte growth factor (HGF), members of the fibroblast growth factor (FGF) family and transforming growth factor beta 1 (TGF-β1) [37]. More recently NRP1 has been shown to interact also with ANGPTL4 (angiopoietin like 4) [47].

Moreover, NRP1 also interacts with other extracellular binding partners that do not belong to growth factors. In fact it was also originally discovered as an adhesion protein on the axons of the developing frog nervous system [62] and later identified in mammals as a receptor for the class 3 semaphorin family (SEMA3), which includes secreted molecules that act as axon guidance cues but can also modulate endothelial function, such as SEMA3A [63,64]. Indeed, NRP1 is required to translate semaphorin cues during neural patterning [65,66].

## 7. NRP1: Expression Pattern and Vascular Function

During development, NRP1 is highly expressed in blood vessels to promote angiogenesis [67]. Accordingly, constitutive NRP1 knockout mice are embryonically lethal due to severe vascular defects in several organs [44,68,69] together with defective remodeling of the cardiac outflow tract and formation of the aortic arch [70]. In particular, we and others have shown that NRP1 is required within the angiogenic endothelium to generate the specialized tip cells that lead vessel sprouts [44,68,69]. Surprisingly, NRP1’s essential role in angiogenesis is only partly explained by its ability to bind VEGFA164 [71,72], with recently identified pathways including NRP1-dependent modulation of both extracellular matrix [45,73] and TGFβ signaling [74]. NRP1 role in outflow tract remodeling is also independent of VEGFA, whereby endothelial NRP1 translates neural crest-derived SEMA3C signals to promote endothelial-to-mesenchymal transition leading to outflow tract septation [75].

**Figure 2:**
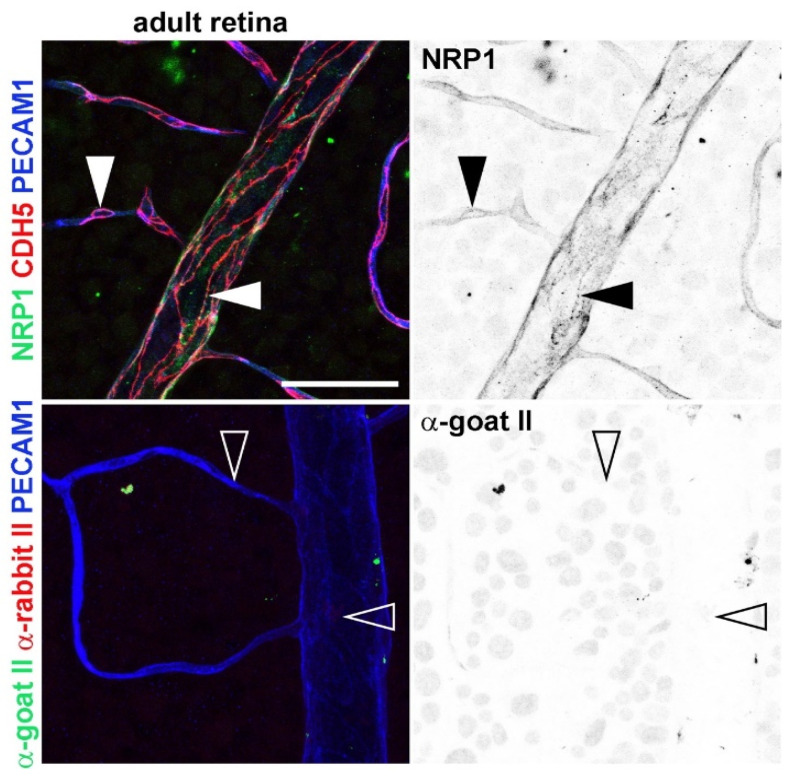
Whole-mount immunostaining of the superficial plexus of adult mouse retina for NRP1, the adherens junction protein CDH5 (VE-Cadherin) and the adhesion molecule PECAM1 (top panels). The panels at the bottom show immunostaining for the same markers but omitting the primary antibody for NRP1 and CDH5. The panels on the right show the green channels as inverted black and white. Arrowheads indicate examples of endothelial junction sites enriched for NRP1 in capillary and venules (top panels) and similar sites in the no primary control (bottom panels). Bar, 50 μm.

More recently, we have shown that NRP1 expression is maintained in the adult quiescent endothelium, including postcapillary venules (Figure 2) [45], where vascular hyperpermeability events mostly occur [8]. Thus, NRP1 concentrated to areas enriched for the adherens junction proteins PECAM1 (platelet endothelial cell adhesion molecule 1) and CDH5 (VE-cadherin) (Figure 2) [45], in agreement with a role for NRP1 in regulating vascular permeability.

## 8. NRP1 and Its Ligands in Vascular Permeability

Since NRP1 is able to interact with multiple ligands and co-receptors, NRP1 can promote a wide range of functions, including the promotion of vascular permeability.

### 8.1. VEGFA Signaling in Vascular Permeability

While VEGFA is best known as an angiogenic growth factor, it was originally described as a vascular permeability factor because it disrupts endothelial barrier function and thereby increases vascular leakage and interstitial pressure [76]. Most studies on the underlying mechanisms of VEGFA-induced vascular hyperpermeability have focused on VEGFA165 alone, or VEGFA164 if in mouse, since it is the most abundant and the most pathological VEGFA isoform [77]. Yet, all VEGFA isoforms have been shown to induce vascular hyperpermeability [45,48,78,79,80].

The tyrosine kinase receptor VEGFR2 has been implicated as the main VEGFA receptor for promoting endothelial hyperpermeability signaling in various organs, including the lung, skin and brain [12,81,82,83,84,85]. The role of the other tyrosine kinase receptor, VEGFR1, in promoting VEGFA-induced permeability remains unclear. Using the Miles assay as an in vivo technique to study vascular hyperpermeability through the proxy measurement of vascular leakage [45,86], two separate studies have shown seemingly contradicting results. On the one hand, loss of VEGFR1 appears to enhance VEGFA-induced permeability, suggesting the receptor mainly functions as a decoy [87], as widely accepted during developmental angiogenesis [88], whilst on the other hand targeting VEGFR1 kinase domain reduces vascular leak in response to VEGFA [89]. Interestingly, uneven apicobasal distribution of VEGFR1 and VEGFR2 in some endothelia, such as in the central nervous system, results in polarized signaling responses to VEGFA, with abluminal VEGFR2 mediating permeability signals while VEGFR1 leads to cytoprotection [84], suggesting that VEGFR2 is more likely to act as the main VEGFA receptor in vascular permeability.

In response to VEGFA, VEGFR2 undergoes dimerization and autophosphorylation at several sites, including the tyrosine (Y) 949 residue (Y951 in human) that is essential to transduce VEGFA signals into increased vascular leakage via sequential phosphorylation of cytoplasmic SRC family kinases (SFKs) and junctional VE-cadherin [85]. Within the SFK family, only SRC (also known as c-Src) and YES1 kinases have been implicated in promoting VEGFA-induced permeability signaling in vivo [90,91], even though recent findings suggest that loss of SRC does not affect endothelial cell-cell adhesion, which is required for vascular integrity maintenance [92]. Instead, the closely related FYN [90] and LYN [93] have been shown to be dispensable for promoting permeability in vitro, with LYN even being implicated in preventing vascular permeability. Future in vivo work deploying cell type-specific null mutations for SRC and YES1 in mice will allow the precise function and relative importance of these two SFKs in VEGFA-induced permeability to be defined.

In order to activate SFKs, the VEGFR2 Y949 phosphosite has been shown to recruit an adaptor molecule, T cell-specific adaptor (TSAd, also known as SH2D2A), which can directly interact with SFKs to translate VEGFA permeability signals [83]. Our recently published work has shown that VEGFA165-SFK activation is additionally regulated by the ABL kinases, ABL1 and ABL2 (also known as ARG). Specifically, VEGFA stimulation activates ABL1 and ABL2 in human ECs in vitro [94] and ABL kinase inhibition or depletion is sufficient to impair the VEGFA165-stimulated activation of SFKs in cultured primary human endothelial cells (HDMECs) [45]. Moreover, ABL kinase activation is essential for VEGFA-induced vascular permeability in the Miles assay [95,96].

Following a distinct VEGFR2-dependent pathway, disassembly of adherens junctions in response to VEGFA can also occur via phosphorylation of AKT1 and subsequent activation of endothelial nitric oxide synthase (eNOS), whereby NO production induces S-nitrosylation of β-catenin that will cause its dissociation from VE-cadherin [97,98]. Other intracellular mediators involved in translating VEGFA permeability signals include Rho GTPases, actin cytoskeleton, focal adhesion kinase (FAK) and cell-matrix adhesion as recently reviewed [9].

### 8.2. NRP1 Role in VEGFA Permeability Signaling

Since it is widely accepted that VEGFA121 does not signal through NRP1 due to the isoform’s low affinity for this receptor, several reports in the last two decades focused on the role of NRP1 in vascular hyperpermeability induced by VEGFA165. Evidence to support a role for NRP1 in VEGFA165-induced vascular hyperpermeability was obtained by genetic studies in which mice lacking endothelial NRP1 expression [45,99] showed reduced intradermal leakage in response to VEGFA164 in the Miles assay. Moreover, a peptide blocking VEGFA164 binding to NRP1 inhibits serum albumin leak in a mouse model of diabetic retinal injury [100], and function-blocking antibodies for NRP1 suppress intradermal vascular leak induced by VEGFA164 injection [101], as well as VEGFA164-induced pulmonary vascular leak [102]. In contrast, other studies argued against an important role for NRP1 in VEGFA-induced vascular permeability, with one study showing that an antibody blocking VEGFA164 binding to NRP1 impaired corneal neovascularization, but not VEGFA164-induced intradermal vascular permeability in mice [103], and another study finding that NRP1 deletion does not impair VEGFA164-induced permeability of retinal vasculature [104]. To conclusively resolve these controversies we recently took advantage of a comprehensive range of mouse mutants to demonstrate an essential contribution of NRP1, which is dependent on its VEGFA164-binding pocket [45,99]. These findings are compatible with a model in which VEGFA164 binding to NRP1 induces complex formation between NRP1 and VEGFR2, whereby VEGFR2 depends on NRP1 to evoke a maximal permeability response to VEGFA164 through ABL-mediated SFK activation [45]. NRP1 closely related family member, NRP2, is instead unlikely to be involved in VEGFA-induced vascular hyperpermeability, as VEGFA165 has a 50-fold lower affinity for NRP2 compared to NRP1 [58], even though direct experimental evidence would be required to prove it.

**Figure 3:**
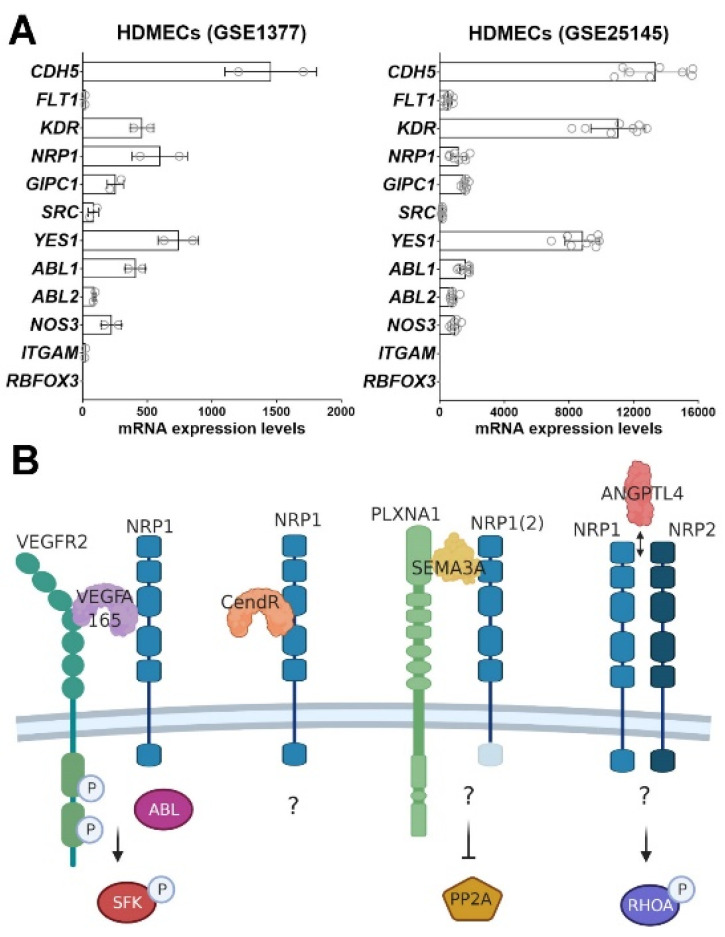
(**A**) Summary of the molecular mediators involved in VEGFA-induced permeability signaling and their relative expression pattern in published transcriptomic data from cultured HDMECs. The GEO identification number for two different microarray studies are indicated on top of the graphs. CDH5 expression is used as a positive control whilst expression of the myeloid-specific (ITGAM) and neuronal-specific (RBFOX3) genes are shown as negative controls. (**B**) Current working models for the early signaling events mediated by NRP1 to transduce vascular permeability signals from different ligands. While the intracellular targets of VEGFA165, SEMA3A and ANGPTL4 signaling convey to the destabilization of EC-EC junction via different pathways to promote paracellular permeability, the permeability route induced by the CendR peptide stimulation of NRP1 has not been definitively explored yet (see text). NRP1 cytoplasmic domain is shown in transparent mode in the SEMA3A pathway because, on the contrary of VEGFA165 and CendR, SEMA3A permeability signaling does not require it, while for ANGPTL4 is not yet known. Even though, each ligand and receptor are known to mostly act as homodimers, for simplicity reasons we represented them as monomers. Created with BioRender (https://biorender.com/, accessed on 19 April 2021).

Recently, we demonstrated that mice lacking the NRP1 cytoplasmic domain displayed less leakage when stimulated with VEGFA164 in the Miles assay [45]; an unexpected result as the cytoplasmic tail of NRP1 lacks kinase activity and does not participate in NRP1 functions during both developmental and pathological angiogenesis [53,105]. Moreover, the cytoplasmic tail of NRP1 promotes the VEGFA-dependent activation of ABL kinases and SFKs [45], which are both essential for translating VEGFA permeability signals (see above) (Figure 3B). NRP1 cytoplasmic domain can therefore discriminate between NRP1 angiogenesis and permeability functions. The only known intracellular interactor of NRP1 is GIPC1 that, upon complex formation of VEGFA, VEGFR2 and NRP1, is recruited to the cytoplasmic tail of NRP1 to traffic the receptor complex into signaling endosomes to promote arteriogenesis [53]. However, mice that lack GIPC1 display normal vascular leakage in response to VEGFA164 [45]. Hence, permeability and arteriogenic VEGFA signaling both rely on NRP1 cytoplasmic domain but can be distinguished by GIPC1 dispensability for VEGFA-induced vascular leakage. Unfortunately, the identity of the NRP1 cytoplasmic domain-binding partner that promotes hyperpermeability remains so far unknown. Further work is therefore still necessary to shed light on a mechanism that, if targeted, may be a useful therapeutic strategy in neovascular disease to reduce VEGFA165-induced edema without compromising vessel growth. 

To recapitulate, Figure 3A shows a summary of the molecular mediators involved in VEGFA-induced permeability signaling and their relative expression pattern in published transcriptomic data from cultured HDMECs [106,107], which are widely used to study endothelial barrier function in vitro [8,108].

### 8.3. C-End Rule Peptides

Like VEGF ligands, most of the known natural proteins or artificially generated peptides with NRP1-binding activity bind through a carboxy (C)-terminal R/KXXR/K minimal sequence motif (X stands for any amino acid); this requirement is called the C-end rule (CendR [101]). All the peptides sharing the same sequence motif bind to the ligand-binding pocket domain in the b1/b2 domains of NRP1 [34,109]. In a combination of both in vitro and in vivo assays, Roth and colleagues recently showed that a tetrameric CendR peptide induces NRP1 accumulation at endothelial cell-cell contacts and vascular leakage [48]. Even though this process is regulated by the NRP1 cytoplasmic domain, the signaling pathway is distinct from the one mediated by VEGFA165, since it does not include activation of VEGFR2, AKT1, p38 (MAPK14), ERK1/2 (MAPK3/1) or FAK (PTK2) [48]. In fact, CendR peptides bind NRP1 to induce vascular permeability independently of VEGFR2 activation [48] (Figure 3B). In agreement with different pathways triggered by CendR peptides versus VEGFA, a previous study has demonstrated that GIPC1 interaction with NRP1 is required for CendR peptide-mediated endocytosis [110], and might similarly be involved in CendR peptide-mediated permeability. Moreover, the authors further suggest that CendR peptide internalization leads to the formation of VVOs [110]. It is therefore possible that CendR peptide-mediated vascular leakage could result from a transcellular route.

### 8.4. SEMA3A

In addition to binding VEGFs, NRPs are co-receptors for members of the semaphorin family. In particular, on top of its original role in axonal guidance, SEMA3A has been widely reported to affect endothelial behavior, including the regulation of vascular barrier, with NRP1 being a key player in the regulation of this pathway. Thus, SEMA3A induces vascular hyperpermeability in a NRP1-dependent mechanism in the mouse Miles assay [45,99] and SEMA3A association with NRP1 induces the loss of the blood-brain [111] or blood-retinal barrier integrity [104].

The VEGFA and SEMA3A permeability pathways have been proposed to diverge, despite their shared NRP1 dependence. The difference between these two pathways involves ligand binding to different extracellular domains of NRP1. Indeed, crystallographic evidence revealed that VEGFA165 and SEMA3A do not directly compete for NRP1, but rather can simultaneously bind to NRP1 at distinct, nonoverlapping sites [112] (Figure 3B). The NRP1 cytoplasmic domain is required for VEGFA-induced SFK activation and vascular leakage while both SFKs and the cytoplasmic tail of NRP1 are dispensable for SEMA3A-induced vascular barrier disruption [45,99]. Moreover, SEMA3A-induced vascular permeability has been shown to require the PLXNA1 transducing receptor to destabilize EC-EC junctions integrity through VE-cadherin serine phosphorylation and internalization [113] (Figure 3B). Mechanistically, stimulation with SEMA3A transiently disrupts the serine/threonine phosphatase PP2A interaction with VE-cadherin, thereby allowing VE-cadherin phosphorylation [113] (Figure 3B).

In complete antithesis with the literature reviewed above, one study reported that SEMA3A can also induce permeability signals by acting via NRP2 and VEGFR1, independently of NRP1, in cultured brain endothelial cells [114]. Even though NRP2 has been shown to bind SEMA3A also in cellular contexts other than ECs [65], among semaphorins, NRP2 is well known to bind preferentially SEMA3F [66]. Contrarily to SEMA3A, SEMA3F inhibited VEGFA-induced vascular permeability in the Miles assays in mice and, at equal doses, SEMA3F protein was as effective as bevacizumab, a VEGFA-neutralizing antibody, in blocking vascular permeability [115]. Since mice lacking *Nrp2* show increased vascular permeability in inflamed ears, the authors suggest that SEMA3F inhibition of vascular permeability might be mediated by its co-receptor NRP2 [115]. Further work will therefore be required to understand the relative significance of NRP1- and NRP2-dependent permeability signals driven by SEMA3A and SEMA3F in ECs.

### 8.5. ANGPTL4

The expression of ANGPTL4, a HIF1-regulated gene product, is increased in the eyes of diabetic mice and patients with DME. ANGPTL4 is a multifunctional circulating protein that undergoes proteolytic processing by membrane proprotein convertases upon secretion. The resulting C-terminal domain (cANGPTL4) appears to have an important role in vascular hyperpermeability [116,117,118,119,120]. However, cANGPTL4 is not able to bind TIE1 or TIE2 (TEK) receptors, which are the cognate receptors for other related angiopoietins, ANGPT1 and ANGPT2 [121]. ANGPTL4 was therefore considered an orphan ligand until a recent study demonstrated that cANGPTL4 is able to bind NRP1 and also NRP2 with similar affinities to VEGFA165. In particular, cANGPTL4 can form a complex with both NRP1 and NRP2 to promote vascular permeability in vivo via RHOA activation [47] (Figure 3B). Interestingly, this study also showed that VEGFR2 is not required for ANGPTL4 promotion of EC permeability [47]. Yet, the exact signaling pathway activated by this ligand in ECs still remains to be elucidated, including the mechanism of cANGPTL4 binding to NRP1, considering that its C-terminus does not follow the C-end rule (see above) and any possible involvement of the NRP1 cytoplasmic domain.

## 9. NRP1 Regulation of Vascular Permeability in Disease

A non-physiological increase in vascular permeability is a common denominator of several diseases. However, since the role of NRP1 in pathological vascular permeability has been mainly studied in preclinical models of neovascular eye diseases and cancer, we will now focus on NRP1 regulation of vascular permeability in these two sets of diseases.

### 9.1. Eye Diseases

The aberrant expression of proangiogenic factors such as VEGFA and other vasoactive mediators can lead to the deterioration of the blood-retinal barrier culminating in the accumulation of interstitial fluid in the macula, which can lead to macular edema, the major cause of severe vision loss in the Western world working population [122]. Diabetes, and more precisely diabetic retinopathy, is a common cause of macular edema, resulting in DME. Different studies have shown that injection of soluble NRP1 is able to reduce retinal vascular leakage in diabetic animals by sequestering either both VEGFA and ANGPTL4 or SEMA3A [47,104]. Interestingly, these different NRP1 ligands are involved in different stages of the DME pathogenesis. For example, SEMA3A expression is robustly induced in the early hyperglycemic stage of diabetes in humans and in a mouse model of type 1 diabetes induced by streptozotocin treatment, raising the possibility that it could represent a valid therapeutic target to stem excessive vascular permeability in DME [104]. Moreover, SEMA3A elevates vascular permeability and contributes to tissue damage also in models of brain ischemia [114].

Edema in DME and AMD can be significantly reduced with anti-VEGFA therapies [123]; however, recent studies in the mouse suggest that global VEGFA blockade in retinal diseases might have detrimental side effects in the long-term. In particular, VEGFA is a survival factor for retinal neurons [124,125], and reducing VEGFA levels in the mouse eye compromises the maintenance of the choroidal vasculature that is essential for photoreceptor health [124,126]. Accordingly, NRP1-based therapeutics might provide an alternative approach for treating vascular leakage in eye disease when anti-VEGFA treatment is not suitable or effective. Moreover, an alternative strategy to prevent ocular vessel leakage may involve targeting both VEGFA and SEMA3A signaling at the same time. Such an approach would involve either 2 separate drugs each specific for one of the 2 pathways or a single molecule able to block both ligand-binding domains in NRP1 or to inhibit a common downstream target.

Instead of targeting the extracellular binding of NRP1 ligands, targeting the NRP1 cytoplasmic domain-mediated signaling pathway was recently suggested. Thus, in a mouse model of choroidal neovascularisation with pathological vascular changes akin to those observed in exudative AMD [125], mice lacking the NRP1 cytoplasmic domain had significantly reduced ocular vascular leakage, while neovascularization was unchanged [45]. A therapeutic strategy targeting the permeability signaling controlled by NRP1 cytoplasmic domain may be particularly useful to selectively treat VEGFA165-induced vascular leak without compromising other VEGFA functions; especially when VEGFA-dependent cytoprotection or the formation of new blood vessels are required, for example in ischemic tissues and not only in the eye.

### 9.2. Cancer

Tumor vasculature usually displays hierarchical disorganization, increased tortuosity, poor perfusion and instability, as well as increased vascular leakage. Since anti-angiogenic strategies have shown some beneficial effects in cancer treatment but to a minor extent than what was expected from earlier preclinical studies, a current trend in the field is to focus instead on the normalization of the tumor vessels, in particular to attenuate their exaggerated permeability. Therapies aimed at targeting NRP1-dependent permeability signals could therefore find an application for this purpose.

Interestingly, Treps and colleagues reported that extracellular vesicles released by glioblastoma cancer cells transport SEMA3A, which enhances vascular permeability through NRP1 independently of VEGFA [127]. Despite its known permeability-inducing property, SEMA3A has also been proposed as a normalizing agent for anti-tumoral treatment [128,129]. To this aim, the authors engineered a mutant version of SEMA3A that cannot interact with NRP1 to prevent vascular permeability, while preserving other desired properties of SEMA3A, such as the repulsion of migrating ECs that promotes blood vessel normalization in vivo and ultimately inhibits tumor growth and dissemination to distant organs [130].

Opposite to eye diseases, whereby vascular permeability is considered a valid therapeutic target only when inhibited, a few teams have proposed to exploit NRP1 pro-permeability properties to promote penetration of co-injected anti-cancer drugs and develop more efficient delivery systems [131,132]. Thus, studies on the permeability-inducing properties of CendR peptides are of fundamental translational importance as such peptides can be exploited to enhance tumor penetration of chemotherapeutic drugs and consequently their efficacy while reducing their side effects [133]. CendR properties have also been combined to those of RGD peptides to generate a tumor-penetrating peptide, iRGD, that homes to tumors by initially binding to αv integrins that are specifically expressed on the endothelium of tumor vessels. iRGD is then proteolytically cleaved in the tumor and, despite losing much of its integrin-binding activity, the truncated peptide gains affinity for NRP1 because of the C-terminal exposure of a CendR motif [134,135]. Moreover, the peptides can be administered either in combination or conjugated to anti-cancer molecules or paramagnetic nanoparticles usable in magnetic resonance imaging to improve tumor homing and penetration [134,135,136,137,138]. These strategies are considered promising applications especially in glioblastoma to enhance the penetration of the blood-brain barrier [139,140].

## 10. Conclusions/Perspectives

NRP1 ability of binding different types of extracellular ligands as well as its involvement in multiple signaling pathways makes it a fascinating pharmacological target, whose blockade or exploitation may be beneficial in diseases associated with vascular leakage or that require improved tissue penetration, respectively. While the existing data already provide extensive insights, further studies are clearly needed to better define the precise effector mechanisms that enable NRP1 to convey disparate signals into the induction of vascular permeability.

The interest in NRP1 targeting further increased following the COVID-19 outbreak. In fact, NRP1 has very recently been shown to serve as an entry factor and potentiate SARS Coronavirus 2 (SARS-CoV-2) infectivity in vitro [141]. By modulating SARS-CoV-2 infectivity as well as the adhesion and permeability of ECs, NRP1 could very well play a role in severe COVID-19 associated with vascular pathologies [142].

## Abbreviations

VEGF	vascular endothelial growth factor
NRP	neuropilin
EC	endothelial cell
DME	diabetic macular edema
AMD	age-related macular degeneration
VPF	vascular permeability factor
JAM	junction-associated molecule
ZO	zona occludens
VE	vascular endothelial
CDH5	cadherin 5
VVO	vesiculo-vacuolar organelle
PLVAP	plasmalemma vesicle associated protein
GIPC1	GAIP-interacting protein C terminus, member 1
VEGFR	vascular endothelial growth factor receptor
FLT1	Fms Related Receptor Tyrosine Kinase 1
FLK1	kinase Insert Domain Receptor 1
PLGF2	placental growth factor 2
PGF	placental growth factor 2
HGF	hepatocyte growth factor
FGF	fibroblast growth factor
ANGPTL4	angiopoïetine like 4
SEMA3	semaphorin 3
TGF-β	transforming growth factor beta
PECAM1	platelet endothelial cell adhesion molecule 1
SFK	Src family of protein tyrosine kinases
SRC	SRC Proto-Oncogene, Non-Receptor Tyrosine Kinase
YES1	YES Proto-Oncogene 1, Src Family Tyrosine Kinase
FYN	FYN Proto-Oncogene, Src Family Tyrosine Kinase
LYN	LYN Proto-Oncogene, Src Family Tyrosine Kinase
HDMEC	human dermal microvascular EC
MLEC	mouse lung EC
MBEC	mouse brain EC
TSAd	T cell-specific adaptor
ABL	Abelson tyrosine kinase
AKT1	RAC-alpha serine/threonine-protein kinase
NOS3, eNOS	nitric Oxyde Synthase 3, endothelial NOS
FAK	focal adhesion kinase
KDR	kinase Insert Domain Receptor
ITGAM	integrin Subunit Alpha M
RBFOX3	RNA Binding Fox-1 Homolog 3
CendR	C-end rule
MAPK	mitogen-activated protein kinases
ERK	extracellular signal-regulated kinases
PLXNA1	plexin A1
PP2A	protein phosphatase 2
HIF1	hypoxia-inducible factor 1
COVID-19	coronavirus disease of 2019
SARS-CoV-2	severe acute respiratory syndrome coronavirus-2
